# Development and Pilot Testing of Text Messages to Help Reduce Sugar-Sweetened Beverage Intake Among Rural Caregivers and Adolescents: Mixed Methods Study

**DOI:** 10.2196/14785

**Published:** 2019-07-30

**Authors:** Maryam Yuhas, Kathleen J Porter, Donna-Jean P Brock, Annie Loyd, Brittany A McCormick, Jamie M Zoellner

**Affiliations:** 1 Department of Public Health Sciences University of Virginia Christiansburg, VA United States

**Keywords:** sugar-sweetened beverages, rural health, text message, mixed-methods, caregivers, adolescents

## Abstract

**Background:**

A high consumption of sugar-sweetened beverages (SSBs) poses significant health concerns, particularly for rural adults and adolescents. A manner in which the health of both caregivers and adolescents can be improved is by developing innovative strategies that target caregivers as the agents of change. Sending text messages through mobile phones has been cited as an effective way to improve behavioral outcomes, although little research has been conducted in rural areas, particularly focusing on SSB intake.

**Objective:**

By targeting rural caregivers, this 2-phase study aimed to (1) understand caregivers’ perceptions and language preferences for SSB-related text messages to inform and refine message development and delivery and (2) evaluate the acceptability of text messages for SSB intake behavior change and examine short-term effects on SSB intake behavior.

**Methods:**

A convergent mixed methods design was used to systematically develop and pilot-test text messages with caregivers in Southwest Virginia. In phase 1, 5 focus groups that included a card-sorting activity were conducted to explore advantages/disadvantages, language preferences (ie, tone of voice, audience, and phrase preferences), and perceived use of text messages. In phase 2, caregivers participated in a 5-week text message pilot trial that included weekly educational and personalized strategy messages and SSB intake assessments at baseline and follow-up. Before the focus groups and after completing the pilot trial, caregivers also completed a pre-post survey that assessed SSB intake, SSB home availability, and caregivers’ SSB-related practices. Caregivers also completed individual follow-up telephone interviews following the pilot trial.

**Results:**

In phase 1, caregivers (N=33) reported that text messages were convenient, accessible, and easy to read. In addition, they preferred messages with empathetic and authoritative tones that provided useful strategies and stayed away from using absolute words (eg, always and never). In the phase 2 pilot trial (N=30), 87% of caregivers completed baseline and 77% completed follow-up assessment, suggesting a high utilization rate. Other ways in which caregivers reported benefiting from the text messages included sharing messages with family members and friends (80%), making mental notes (57%), and looking back at messages as reminders (50%). Caregivers reported significant improvements in home environment, parenting practices, and rulemaking around SSB (*P*=.003, *P*=.02, and *P*=.04, respectively). In addition, the frequency of SSB intake among caregivers and adolescents significantly decreased (*P*=.003 and *P*=.005, respectively).

**Conclusions:**

Spending time in the formative phases of text message development helped understand the unique perspectives and language preferences of the target population. Furthermore, delivering an intervention through text messages has the potential to improve caregiver behaviors and reduce SSB intake among rural caregivers and adolescents. Findings from this study were used to develop a larger bank of text messages, which would be used in a future study, testing the effectiveness of a text message intervention targeting SSB intake–related caregiver behaviors.

## Introduction

### Background

Sugar-sweetened beverages (SSBs) pose significant health concerns because of the excessive amounts consumed across the United States [1]. Several systematic reviews and meta-analyses have identified health risks associated with increased SSB consumption, including obesity, cardiovascular disease, and obesity-related cancers [2-4]. High consumption is even more concerning for populations in which these diseases are known to be disproportionately high, such as rural adults and adolescents [5-8]. The rurality status has been associated with an increased likelihood of drinking more than 3 cans of SSBs per day [9,10]. Developing strategies to reduce SSB intake that target caregivers as the agents of change in the home could be a promising way to improve SSB intake behaviors within families.

Many studies have found that caregivers are significant influencers of adolescents’ dietary habits through their role modeling of behaviors, parenting practices, and management of the home environment [11,12]. In rural areas, multilevel interventions targeting adolescent SSB consumption, which also address caregivers’ influence, are substantially lacking [13]. Possible reasons for this are the multitude of barriers faced by rural residents, such as lack of transportation, geographical dispersion, and reduced health services that make it difficult to access disease prevention programming [14]. Consequently, there is a need to develop and test scalable strategies that overcome these barriers while reaching and engaging rural caregivers.

One such strategy that is gaining momentum is the use of text messaging for behavior change. Mobile phone and text message use are rising rapidly in the United States, with 95% of the adult population owning a mobile phone with text message capabilities [15,16]. In rural areas, 91% of adults have text messaging–capable phones, and 65% of these are smartphones, which can go beyond simple text messages [16]. Furthermore, text message use is high in low socioeconomic populations and those with poorer health, thus making text message a prime modality for health interventions in rural areas [17].

Importantly, systematic reviews have found that text message–delivered interventions are effective in producing positive behavioral outcomes [17,18]. Specifically, preliminary studies indicated that text messages had promise in delivering SSB strategies [19,20]. A small study that used text messages to modify SSB intake behaviors found that attrition rates were lower and adherence to self-monitoring was significantly higher when compared with control groups that did not receive text messages [19]. Although this study shows promise, it did not find significant changes in SSB intake behaviors and has limited generalizability to rural areas. In fact, there are no known published studies that use text messages targeted at rural caregivers to reduce SSB intake among both caregivers and adolescents.

### Theoretical Rationale

Although interventions using text messages for health behavior change are on the rise, few have documented theoretical rationales [18]. Similar to any intervention, it is important to ground the content of text messages in behavioral theory, such as the Theory of Planned Behavior, to optimize the likelihood of promoting behavior change [21,22]. With the brief nature of text messages, it may also be important to consider a language theory in the development of these short messages. Linguistic theory can provide insight into how the language and *paralanguage* (ie, the nonlexical features) of the messages play a role in the overall meaning and effect [23]. These features of the messages are elements such as the tone of voice and message phrasing [23]. These issues become increasingly important when delivering health education messages that are limited in characters, such as the 160-character limit for text messages. A study by Pollard et al explored the tone of voice and content of text messages aimed at changing dietary behaviors in young adults and found that offering substitutes and empathetic tones were most likely to motivate behavior change [24]. As suggested by linguistic theory, spending time in formative phases to understand these features specific to the target population might increase the effectiveness of the text messages to change SSB intake behaviors [25].

### Objectives of This Study

Targeting rural caregivers, the objectives of this 2-phase study were to (1) understand caregivers’ perceptions and language preferences for SSB-related text messages to inform and refine message development and delivery and (2) evaluate the acceptability of text messages for SSB intake behavior change and examine short-term effects on SSB intake behaviors. In phase 1, this study explored advantages/disadvantages, language preferences (ie, tone of voice, like and disliked words, and target audience), and perceived use of text messages through focus groups and card-sorting activity. In phase 2, a text message pilot trial was conducted to further assess acceptability and examine the effects of a text message pilot trial on SSB intake and behaviors of caregivers and their adolescents. Findings from this study will inform the development of a larger scale multilevel intervention targeting SSB intake among adolescents and their caregivers.

## Methods

### Design

**Figure 1:**
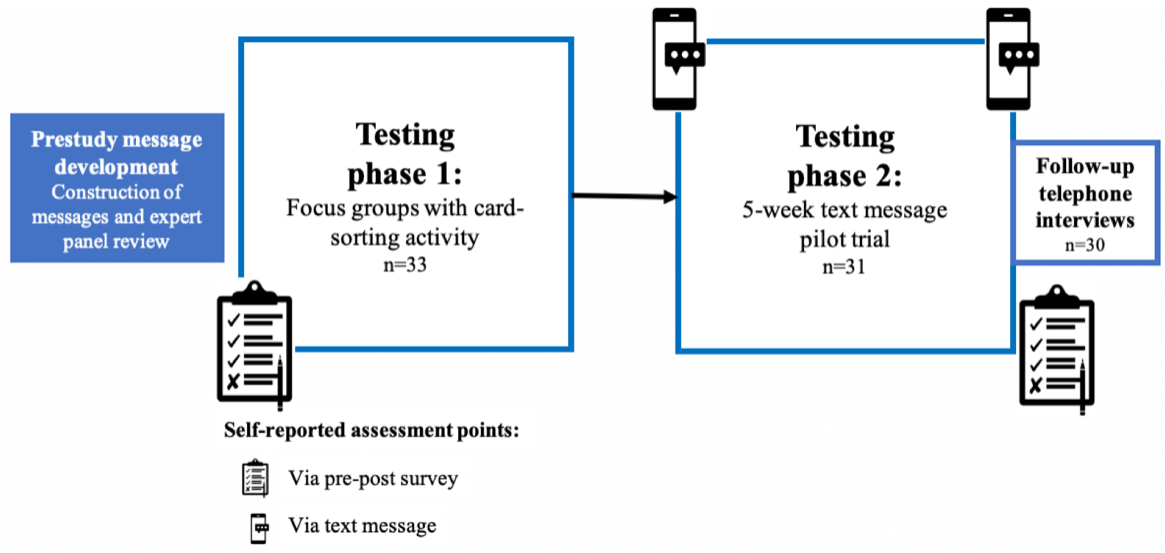
Study overview.

This formative study took place from August 2017 to August 2018. A convergent mixed methods design [26] was used to systematically develop and pilot-test text messages with caregivers (Figure 1). The 2 phases of testing included focus groups with a card-sorting activity and a 5-week text message pilot trial. A pre-post survey was conducted at the beginning of the focus group and the end of the pilot trial. In addition, individual follow-up telephone interviews were conducted with caregivers following the pilot trial.

This study was approved by the Institutional Review Board at the University of Virginia. Caregivers reviewed and signed an informed consent before participating in any study activities. Caregivers received a US $25 gift following the focus group and another US $25 gift card after completing the follow-up telephone interview.

### Participants

To be eligible, caregivers had to be aged at least 18 years, have a child in grades 5 through 8, speak English, and own a mobile phone with text messaging capabilities. Recruitment took place in 3 counties across Southwest Virginia, part of central Appalachia: Tazewell, Wise, and Montgomery. These counties have a rurality status of 7, 5, and 3, respectively, on the rural-urban continuum codes (ie, 1=metro/urban, 9=nonmetro/very rural) [27,28]. A total of 49 caregivers were screened and eligible. Of these, 16 caregivers were either unable to be reached or had a conflict during the time the focus group was held. Overall, 33 caregivers were reached and agreed to attend the focus group. Most of the caregivers were female (85%), white (97%), and had an income >US $55,000 per year (76%). Around 49% were college graduates, 30% had completed graduate school, 12% had completed some college, and 9% had completed high school only. Of these 33 caregivers, 31 (31/33, 94%) participated in the pilot trial and 30 (30/33, 91%) were reached for the follow-up interview.

#### Text Message Development

In the development process, the research team crafted a sample set of text messages by adapting content used in a previous trial grounded in the Theory of Planned Behavior that aimed to reduce SSB consumption in rural adults [29]. For this study, the adapted messages comprised 2 types, educational and strategy messages, both of which aimed to reduce SSB intake. Educational messages contained facts about SSBs, such as what is considered an SSB and health risks of excessive SSB intake. Strategy messages included tips caregivers could use to help reduce SSB intake. In total, the research team developed 7 messages: 4 educational messages and 3 strategy messages. Each message was written in 3 different tones of voice adapted from Pollard et al (ie, authoritative, empathetic, and catchy) [24] and targeted toward 3 different audiences (ie, caregiver, adolescent, and family) for a total of 9 different versions of each message (n=63). The length of each message was kept to 160 characters to stay within the maximum amount of text that can be sent to most mobile telephones. See Table 1 for definitions and example messages.

**Table 1:** Example educational and strategy messages by varying tones of voice and target audience used in testing phases (these messages are the revised versions modified after face validity testing by the expert panel).

Message type and target audience		Authoritative tone: tone conveys a commanding, all-knowing voice and gives the readers information to act on	Empathetic tone: tone conveys that the readers’ struggles are understood, and then asks the readers to act on the information	Catchy tone: tone uses pleasing, rhyming, and easy to remember words to give the readers information to act on
**Educational message: recommendations for sugary drinks**				
	Caregiver-focused	Research says adults should only drink less than 8 oz or 1 small cup of sugary drinks/day, and kids should have 0 oz! Think about where you can cut back.	We know it’s hard to cut back & most people drink too much sugar. Adults should drink <8 oz & kids should have 0 so start by figuring out how much you drink.	Drink less, live more, throw sugar out the door! Limiting sugary drinks to 8 oz for adults can lead to a long and healthy life!
	Adolescent-focused	Research says adults should only drink less than 8 oz or 1 small cup of sugary drinks/day, and kids should have 0 oz! Think about where your child can cut back.	We know it’s hard for your kid to cut back their sugary drinks. Adults should drink <8 oz & kids should have 0. Start by figuring out how much they drink.	Drink less, live more, throw sugar out the door! Helping your kids stop drinking sugary drinks can lead to a long and healthy life for them.
	Family-focused	Research says adults should only drink less than 8 oz or 1 small cup of sugary drinks/day, and kids should have 0 oz! Think about where your family can cut back.	We know it’s hard for your family to cut back their sugary drinks. Adults should drink <8 oz & kids should have 0. Start by figuring out how much they drink.	Drink less, live more, throw sugar out the door! Limiting sugary drinks to 8 oz for adults, and 0 for kids can lead to a long and healthy life for the whole fam.
**Strategy message: bringing alternatives on the go**				
	Caregiver-focused	Stay on track when you’re on the go. Sugary drinks are everywhere, so always remember to pack your favorite non-sugary drink so you don’t slip up.	We know it’s hard to stay on track when you’re on the go. There may be sugary drinks where you go. Pack your favorite non-sugary drink so you don’t slip up!	Don’t slip on your trip! Make sure to carry your favorite non-sugary drink when you leave the house to help stay on track.
	Adolescent-focused	Make sure your child stays on track when on the go. Sugary drinks are everywhere. Always pack their favorite non-sugary drink so they don’t slip up.	We know it’s hard to stay on track when on the go. There may be sugary drinks where your child goes. Pack their favorite non-sugary drink so they don’t slip up!	Don’t let your child slip on their trip! Make sure they carry their favorite non-sugary drink when they leave the house to help keep them on track.
	Family-focused	Make sure your family stays on track when on the go. Sugary drinks are everywhere. Always pack their favorite non-sugary drink so they don’t slip up.	We know it’s hard to stay on track when on the go. There may be sugary drinks where your family goes. Pack their favorite non-sugary drink so they don’t slip up!	Don’t let your family slip on their trip! Make sure they carry their favorite non-sugary drinks when they leave the house to help keep them on track.

Next, the research team sent the messages to an expert panel (n=15) comprising registered dietitians, PhD, and/or graduate level behavioral health researchers to assess the face validity of the messages (ie, intended tone of voice conveyed by messages). The expert panel categorized text messages correctly 67% of the time and provided qualitative feedback regarding areas for improved clarity. The prominent finding that emerged from this panel was to create more distinction between authoritative and empathetic tones. These modifications were made to improve face validity of messages before starting the study.

### Caregiver Pre-Post Survey

Caregivers completed a pre-post survey that assessed demographics, SSB intake, SSB home availability, and caregiver SSB-related practices twice: at the beginning of phase 1 during the focus group and after phase 2.

#### Demographics

Participants reported gender, year of birth, race/ethnicity, education, and income. Race was reported across 5 categories and ethnic background was categorized as Hispanic or non-Hispanic. Education was reported across 6 categories ranging from completion of grades 0 to 8 to graduate school. Income level was reported on 12 categories ranging from <US $5000 to >US $55,000.

#### Caregivers’ Sugar-Sweetened Beverage Intake

An abbreviated version of the validated 15-item beverage intake questionnaire (BEVQ-15) was used to assess SSB intake [30]. The BEVQ-15 includes questions that assess frequency and amount of individual SSBs, including sweetened fruit drinks, soda, sweetened tea, sports and energy drinks, and coffee with cream and/or sugar. Using standardized scoring procedures, the frequency was recoded to ounces per day and calories per day for each SSB.

#### Home Availability of Sugar-Sweetened Beverages

Caregivers reported home availability of individual SSBs on a 5-point Likert scale from *all the time* to *never*, taken from the instrument developed by van de Gaar et al [31]. Responses were reverse coded so that *0* would reflect *never* and *5* would reflect *all the time* and were recoded onto a continuous scale. Availability of each type of SSB was then averaged to obtain availability of all SSBs reported.

#### Caregivers’ Sugar-Sweetened Beverage–Related Practices

Items involving caregivers’ SSB-related practices were obtained from the instrument developed by van de Gaar et al [31]. These included parenting practices toward the adolescent’s SSB intake (4 items), rules at home around the adolescent’s SSB intake (2 items), and role modeling of the caregivers’ SSB intake behaviors (1 item). The items around parenting practices included questions about how often the caregivers monitor the adolescent’s intake, if the adolescent is allowed to drink SSBs whenever he/she wants, if the adolescent receives an SSB when he/she asks for it, and if the caregivers buy the adolescent SSBs when he/she asks for it. The role modeling item asked 1 question around how often the caregivers drink SSBs with the adolescent and how often they drink SSBs in total. Items were assessed on a 5-point Likert scale, with the exception of the 2 items around rules at home with their adolescent, which was reported as yes or no [31]. All Likert-type responses were recoded onto a continuous scale for analysis.

### Phase 1: Focus Groups With Card-Sorting Activity

In the first phase of testing, a trained moderator and comoderator led 5 focus groups using established methods [32]. Each focus group lasted approximately 2 hours, comprised 4 to 9 caregivers, and was audio recorded. At the start of the focus group, caregivers completed a survey (caregiver pre-post survey described above). Then, using a semistructured focus group guide, the moderator elicited caregivers’ thoughts around text message advantages/disadvantages, language preference, and perceived use of text messages for changing SSB intake behaviors.

Participants also completed a card-sorting activity [33] to understand specific language preferences: (1) tone of voice preferences, (2) audience preferences, and (3) liked and disliked words and phrases. Each caregiver was given 3 sets of message cards: 2 educational and 1 strategy message (n=27), a sorting mat, and a pink and green highlighter. Participants were first instructed to sort the cards into 3 piles: liked, disliked, and neutral. Next, caregivers went through their separated piles and used a green highlighter to highlight words and phrases liked and pink highlighter for those disliked. Participants were given the option to write comments on the cards and/or write new text messages. Within the focus groups, message sets (n=7, each set had 9 versions of the same message) were randomly distributed, so that each educational and strategy message set was tested at least once. Afterward, message sets were randomly repeated as needed until thematic saturation was met after 5 focus groups.

#### Phase 1: Data Analysis

Notes and transcriptions were qualitatively analyzed using an inductive approach [34]. First, the primary moderator reviewed the transcripts and took notes to summarize the focus groups. These notes were used to develop the codebook by identifying broad categories (eg, advantages to text messages) and codes within the categories (eg, timing and accessibility). Second, 2 additional coders independently reviewed notes and transcripts and identified meaning units that corresponded to the codes. Categories and codes were reviewed to allow for additions, merging overlap, and removal, as needed. Finally, the 3 reviewers met to resolve discrepancies and gain consensus.

Frequency statistics were used to understand preferences for the tone of voice, audience, and liked and disliked phrases. The card-sorting activity was analyzed in 3 steps. First, the cards were coded with unique identifiers that represented the tone of voice and target audience for each message (eg, a catchy tone geared toward the family would have the code *CaF*). Second, the research team used these codes to tally how many cards fell into the liked, disliked, and neutral categories. For example, if a participant sorted a *CaF* card into a *liked* pile, 1 tally would be marked for the catchy tone and another one for the family audience, within their respective liked column. Educational and strategy messages were analyzed separately to account for preference differences by the type of content. Finally, to analyze liked and disliked words and phrases, the research team used the highlighting on the cards, which was completed after the sorting portion of the activity. Regardless of how the cards/messages were initially sorted into *liked*, *disliked*, or *neutral* piles, participants could still use highlighters (liked and disliked words and phrases) on all cards in each pile. Findings from Phase 1 informed text messages used in the phase 2 pilot trial.

### Phase 2: 5-Week Text Message Pilot Trial

In the second phase of testing, caregivers from the focus groups participated in a 5-week text message pilot trial (see Multimedia Appendix 1 for user experience). The first week of this trial introduced the program, reminded caregivers what counted as an SSB, and delivered the baseline self-reported assessment point via text message. This baseline text message assessment first assessed caregivers’ intake and then adolescents’ intake using a 1-item question adapted from the BEVQ-15 to quickly assess the frequency of all SSB intake for caregiver and adolescent over the past week [30]. Caregivers reported from 7 categories: less than 1 time, 1 time, 2 to 3 times, 4 to 6 times, every day, 2 per day, or 3 or more per day. Responses were recoded to a continuous scale by dividing each response by 7 to reflect a frequency per day.

**Figure 2:**
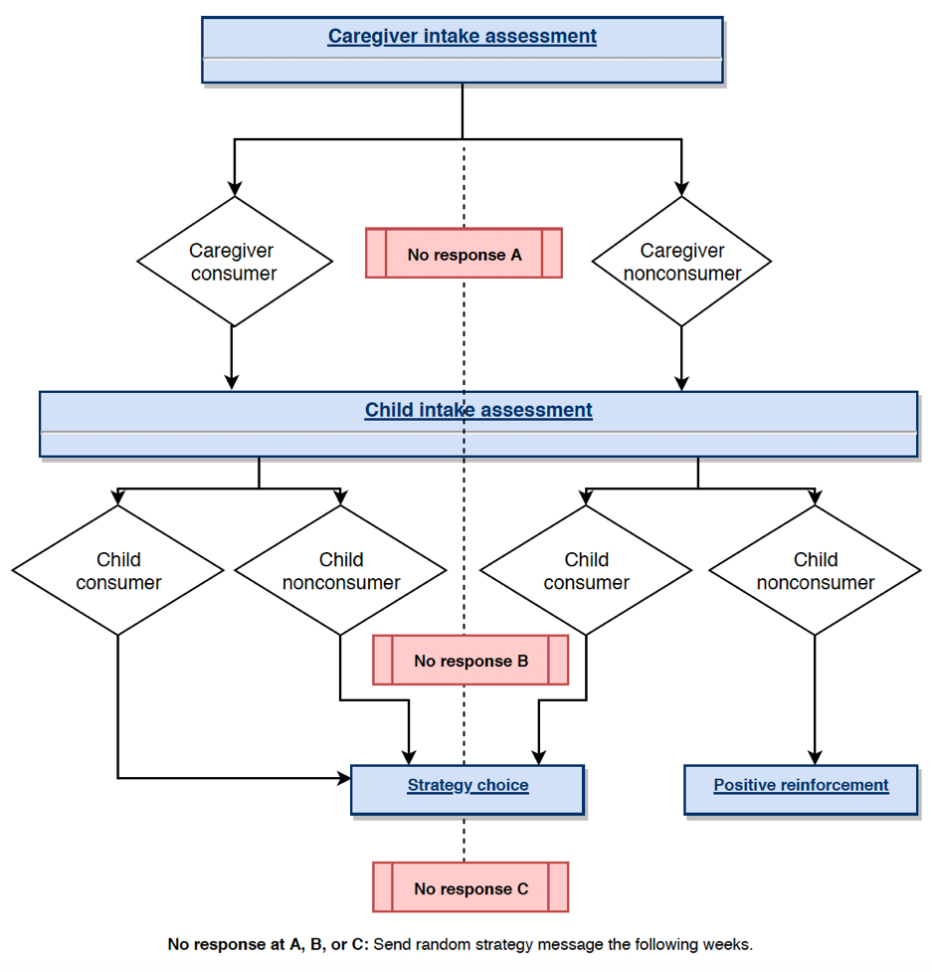
Decision tree for the caregiver text message–based assessment.

Following this, the text message software used caregiver and adolescent intake data to separate caregivers into 4 categories based on SSB consumption patterns (see Figure 2). The consumption category impacted the content of the third message of the assessment. If either were an SSB consumer, the third assessment question was an option to select a personalized strategy (ie, tasty alternatives, breaking your habit, home and shopping tips, and parenting tips). If neither the caregivers nor the adolescents were SSB consumers, caregivers received positive reinforcement (eg, *Congrats on drinking little to no sugary drinks! Keep up the good work.*) Or were given the opportunity to respond with strategies they used with their own families (ie, *Other families could use your help! What types of things do you do as a parent to help your child drink less (or no) sugary drinks?*).

Over the next 3 weeks, caregivers were sent 2 text messages per week: an educational message and a random strategy message from the category chosen (or positive reinforcement). The last week, caregivers received an educational message followed by the final self-reported assessment point via text message. At this last assessment, caregivers were able to choose 1 last strategy message based on consumption patterns. This message was then displayed immediately after they made their strategy selection. The research team collected process data on text message–based assessment response rates, changes in consumer type, and changes in strategy selection from baseline to final assessment.

#### Phase 2: Data Analysis

Quantitative analyses were performed using SPSS statistical analysis software (version 25.0). For data obtained from text messages, frequencies were used to analyze assessment response, consumer category, and personalized strategy choice rates. For both data obtained from text messages and pre-post surveys, descriptive statistics, including means and standard deviations, and paired t tests were used to assess changes in SSB intake, home availability, parenting practices, and role modeling behaviors. Cohen d effect sizes for paired samples were calculated. A McNemar test was used to assess the difference in the proportion of caregivers reporting yes versus no to making rules around SSBs and phi effect sizes were calculated for these 2 items.

#### Follow-Up Telephone Interviews and Data Analysis

Following the text message pilot trial, caregivers also received a follow-up phone call. Research staff trained in qualitative methods conducted the phone calls using semistructured, open-ended questions with probes to reevaluate language preference, perceptions, and overall acceptability of the text messages. The interviews were audio recorded and the interviewer also took notes during these phone calls. Each call lasted between 15 and 20 min. For qualitative findings from the postinterviews, interviewer’s notes were qualitatively analyzed using an inductive approach and quantified across the caregivers [34]. Although the postinterviews were audio recorded, detailed notes of the interviewer provided sufficient information for coding. Only representative quotes were transcribed from audio recordings.

## Results

### Phase 1: Focus Groups With Card-Sorting Activity

#### Semistructured Discussion

Main categories that emerged from the focus groups were advantages and disadvantages of using text messages for SSB intake behavior change, liked and disliked language and features of text messages, and thoughts around best practices to increase text message use among caregivers (eg, personalization, completing assessment via text message, and timing and frequency). Multimedia Appendix 2 illustrates the categories and codes that emerged from the focus groups and sample quotations that represent the codes.

Some of the common advantages identified included that text messages were convenient because of the timing, more accessible than other means of communication, such as fliers or emails, short and easy to read and understand, and are supported by most cellular plans in this region. On the contrary, caregivers felt that some of the disadvantages to text messages were some people might not have text message–capable devices, poor coverage or service areas, and some may be using temporary phones or phone numbers.

Regarding liked and disliked language and features of text messages, some of the top liked responses included messages that contained memorable phrases, used a family approach or sparked discussion with family, provided useful information and solutions to drinking less SSBs, and were phrased encouragingly. Some of the top disliked features included messages that told them what to do without providing any useful strategies, made caregivers feel judged as a parent/caregiver, used symbols that may be hard to interpret, used condescending and demeaning tones, made assumptions about their drinking habits, or used absolute words, such as never, always, or only.

Participants also identified best practices to increase text message use among caregivers. Some caregivers felt that personalizing with the caregiver and/or adolescent name might grab attention, but some others also felt personalization was unnecessary and would not make a difference in their behavior. Participants also felt that doing assessments to check in on caregivers’ and adolescents’ SSB intake would be helpful in reaching their goals; however, the response quality might be poor. Most caregivers preferred or felt that most caregivers would benefit the most from the text messages delivered at a time when they were with the adolescent, such as after school. Some others felt that delivering messages at the start of the day, week, or month would be preferred because that was when most people set goals. Finally, most caregivers agreed that 1 to 2 times per week was a good frequency to receive text messages.

#### Card-Sorting Activity

**Figure 3:**
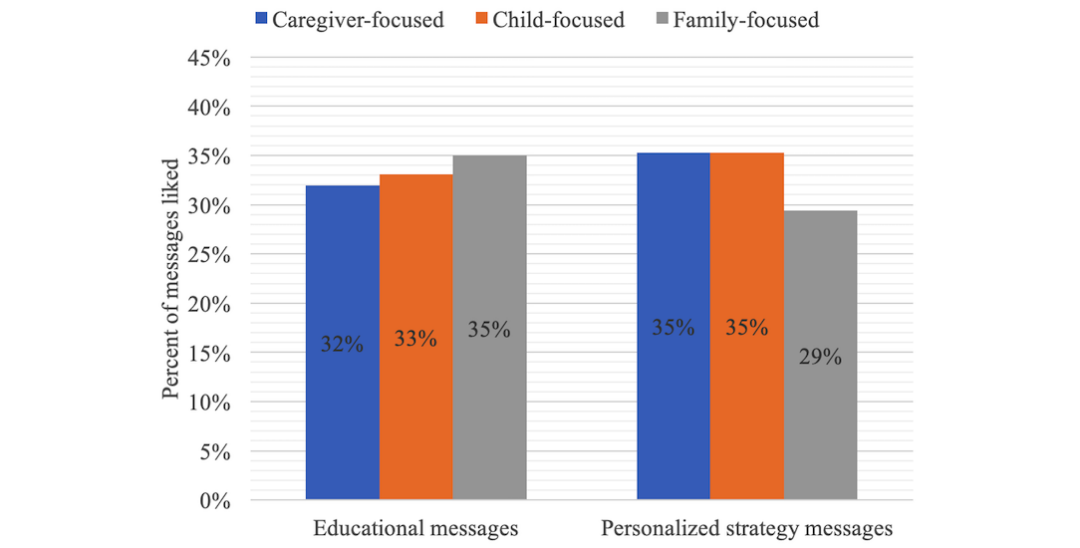
Caregivers’ target audience preferences for educational and personalized strategy text messages related to changing sugar-sweetened beverage intake behaviors.

**Figure 4:**
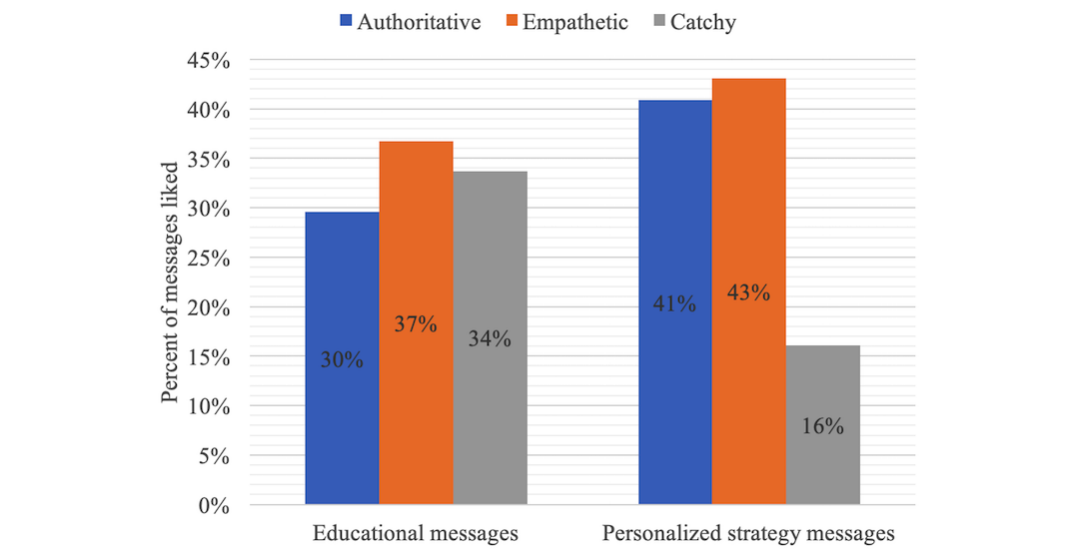
Caregivers’ tone of voice preferences for educational and personalized strategy text messages related to changing sugar-sweetened beverage intake behaviors.

Data analyses revealed that there was no strong preference for messages framed for a particular target audience (Figure 3). Preference for caregiver-, child-, and family-focused messages were relatively evenly distributed at 29% to 35%. However, some tone of voice preferences emerged (Figure 4). Of all educational messages that were liked, 37% were empathetic, 34% were catchy, and 30% were authoritative. Of all personalized strategy messages that were liked, 43% were empathetic, 41% were authoritative, and 16% were catchy (Figure 4). Some common words disliked included absolute words, such as only, always, and never, and commanding words and phrases, such as stop or don’t be tricked. Liked words included positive actions, such as practice and help. Changes were made to messages based on recommendations from semistructured discussions and card-sorting activity before moving into the pilot trial.

### Phase 2: 5-Week Text Message Pilot Trial

#### Text Message Process Data

Of the 31 caregivers, 27 (27/31, 87%) fully completed the text delivered baseline assessment (ie, answered all 3 text message questions on caregivers’ SSB intake, adolescents’ SSB intake, and personalized strategy choice; Table 2). There were also 3 partial completers and 1 nonresponder at baseline. At follow-up, 24 of 31 (24/31, 77%) caregivers fully completed the 3 text message assessment questions and there were 2 partial completers and 5 nonresponders.

At baseline, 19 of 27 (19/27, 70%) caregivers started in the caregiver consumer/adolescent consumer category, but at follow-up, only 8 of 27 (8/27, 33%) were categorized into this group. At follow-up, most caregivers were categorized into the caregiver nonconsumer/adolescent consumer bucket (11/24, 46%). When given the choice of strategy, home and shopping tips was the top choice at both baseline and follow-up (about 45%). The other strategies chosen were relatively evenly distributed, ranging from 8% to 17%. Also, 65% (15/23) of the caregivers changed their strategy from baseline to follow-up (Table 3).

**Table 2:** Text message–based assessment process data: changes in the sugar-sweetened beverage consumption category and changes in personalized strategy choices.

Process data variable		Participants at baseline (n=27)^a^, n (%)	Participants at follow-up (n=24)^a^, n (%)
**Caregiver and adolescent sugar-sweetened beverage intake category^b^**			
	Caregiver consumer/adolescent consumer	19 (70)	8 (33)
	Caregiver consumer/adolescent nonconsumer	1 (4)	1 (4)
	Caregiver nonconsumer/adolescent consumer	3 (11)	11 (46)
	Caregiver nonconsumer/adolescent nonconsumer	4 (15)	4 (17)
**Chosen personalized strategy^c^**			
	Home and shopping strategies	12 (44)	11 (46)
	Parenting strategies	4 (15)	3 (12)
	Strategies to find tasty alternatives	3 (11)	2 (8)
	Strategies to break habit	4 (15)	4 (17)
	Positive reinforcement or qualitative response	4 (15)	4 (17)

^a^Only considers caregivers that fully completed both baseline and follow-up assessments. At baseline, there were 3 partial completers and 1 nonresponder. At follow-up, there were 2 partial completers and 5 nonresponders. Participants were considered partial completer if they did not respond to all 3 assessment questions and if missing data were not considered in the calculations for changes in consumption category and personalized strategy choice.

^b^Categories were assigned based on responses to assessment. Caregivers and adolescents were considered consumers if SSB intake was ≥2 to 3 times per week.

^c^Caregivers who were consumers or had an adolescent that was a consumer were given the choice between strategies. Nonconsumers received positive reinforcement messages or were asked for some tips they would give other families.

**Table 3:** Sugar-sweetened beverage intake and behavior change from text message–based assessment and pre-post survey (n=29^a^).

Variable			Baseline	Follow-up	Effect size, Cohen *d*	Test statistic, paired *t* tests	*P* value
**Text message–based assessment: caregiver and adolescent intake^b^, mean (SD)**						
	Caregiver SSB^c^ intake frequency (times/day)		0.60 (0.53)	0.22 (0.40)	0.82	3.241	.003
	Adolescent SSB intake frequency (times/day)		0.77 (0.70)	0.46 (0.41)	0.54	3.103	.005
**Pre-post survey: caregiver intake only^b^, mean (SD)**						
	SSB intake frequency (times/day)		1.26 (1.25)	0.85 (0.88)	0.38	2.435	.02
	SSB intake (fl oz/day)		17.70 (28.73)	9.60 (9.64)	0.38	1.633	.12
	SSB intake (kcal/day)		184.42 (252.53)	105.83 (110.71)	0.40	1.813	.08
**Pre-post survey: SSB availability in the home^d^, mean (SD)**						
	Total SSBs		1.90 (0.70)	1.47 (0.74)	0.60	3.266	.003
	Coffee w/cream and/or sugar		1.86 (1.33)	1.21 (1.26)	0.50	3.732	.001
	Soda		2.25 (1.43)	1.71 (1.36)	0.39	2.737	.01
	Sweetened tea		1.46 (1.23)	1.07 (1.21)	0.32	2.499	.02
	Sports/energy drinks		1.57 (1.60)	1.18 (1.72)	0.24	1.036	.31
	Sweetened fruit drinks		2.40 (1.17)	2.27 (1.15)	0.11	0.486	.63
**Pre-post survey: caregiver SSB-related practices^e^**						
	Parenting practices, mean (SD)		3.20 (0.8)	3.45 (0.54)	0.37	−2.519	.02
	Role modeling, mean (SD)		2.98 (0.7)	3.17 (0.54)	0.30	−1.516	.14
	**Rules^f^, n (%)**					
		Are there rules in your home about how many sugary drinks your child can drink?	17 (58.62)	24 (82.76)	0.36^g^	4.000^h^	.04
		Are there rules in your home about when your child can drink sugary drinks?	20 (68.97)	23 (79.31)	0.39^g^	0.571^h^	.45

^a^A total of 29 responses were analyzed; however, sample sizes fluctuated between variables because of missing responses.

^b^SSB intake was reported on a 7-point scale from <1 time to 3 or more per day. Responses were recoded to a continuous scale by dividing each response by 7 to reflect a frequency per day. Using standardized scoring procedures, the frequency was recoded to ounces per day and calories per day for each SSB.

^c^SSB: sugar-sweetened beverage.

^d^Reported on a 5-point scale and coded so that 0=never available and 4=available all the time.

^e^Reported on a 5-point scale and coded so that a higher number represents a more positive behavior that would lead to reduced adolescent SSB intake.

^f^Caregivers reported as yes/no. Reported in table as percentage that reported yes so that a higher number represents a more positive behavior.

^g^Effect sizes for the 2 rule making variables are reported as phi.

^h^Changes analyzed for the 2 rule making variables were analyzed using McNemar test.

#### Text Message–Based Assessment

Paired t test analyses of the caregiver-reported text message–based assessments found that both caregivers (*P*=.003) and adolescents (*P*=.005) significantly reduced their frequency of SSB intake per day and effect sizes were medium to large (Table 3).

#### Caregiver Pre-Post Survey

As further illustrated in Table 3, pre-post survey data found that caregivers significantly reduced their frequency of SSB intake per day (*P*=.02). Caregiver changes in SSB calories and fluid ounces per day were not significant. Availability of total SSBs in the home also significantly decreased (*P*=.003). When analyzed by individual SSBs, home availability of coffee with cream and/or sugar (*P*=.001), soda (*P*=.01), and sweet tea (*P*=.02) also each significantly decreased, yet sports drinks and sweetened fruit drinks did not change. Caregiver’s parenting practices significantly improved toward encouraging adolescent behaviors that promote reduced SSB intake (*P*=.02). Role modeling, however, was not significant. Related to parenting rules on SSB intake, rules for when adolescents can have sugary drinks significantly increased (*P*=.04), yet rules for how many sugary drinks an adolescent can have did not significantly change. As shown, effect sizes varied across variables.

### Follow-Up Telephone Interview

After completing the pilot trial, the majority of the caregivers (25/30, 83%) reported liking all the messages and stated high acceptability of receiving an SSB intervention through text messages. Some statements made by caregivers about the acceptability of the trial included:

It was encouraging and informative,

I didn’t realize how much I drank until I joined your program,

and

It was a good way for me to start thinking about a plan.

Most caregivers (28/30, 93%) reported that the number of messages sent was a good amount and would have accepted more than 2 messages per week. A stronger preference for receiving messages in the evening time also emerged after the pilot trial. Some of the ways that caregivers used the messages included making mental notes (17/30, 57%), sharing messages with family members, friends, and coworkers (24/30, 80%), and looking back at messages as reminders (15/30, 50%).

All 30 caregivers who completed the follow-up interview reported that the messages were beneficial to their family. Caregivers stated benefits, such as “It gave me more ammunition as a parent,” and “It’s now at a point where we are discussing the issue and consciously thinking about our choices.” Of these, 87% (26/30) reported making actual changes around SSB intake behaviors, such as changing parenting practices (eg, made rules around when and how many SSBs their adolescent can have, increasing the adolescent’s access to water and healthy alternatives), decreasing home availability of SSBs, increasing communication around making healthy drink choices, reducing SSB intake for both the caregiver and adolescent, and creating a general, constant awareness of their intake. The 2 caregivers that reported no changes were made, stated that they were either maintaining their intake or are now planning to make some changes.

## Discussion

### Principal Findings

This is the first known study to evaluate text messages targeted to caregivers to reduce SSB intake in both caregivers and adolescents in a rural setting. Findings from this multiphase mixed methods approach suggest that texting is an acceptable way to deliver educational, strategy, and assessment messages to change SSB intake behaviors in rural populations. In addition, there are unique linguistic perspectives of rural caregivers to take into consideration when designing behavior change messages that may help improve SSB intake behaviors. These include tone of voice with attention to the words, phrases, and other language and features preferred by the target population. This formative study provides a framework for future research involving the development and testing of text messages targeted at SSB intake and other health behaviors.

### Advantages and Disadvantages

Text messaging has several advantages that make it an appealing modality for health promotion and intervention delivery in rural areas. These advantages include low implementation cost, convenience of accessing messages, and increasing reach to those who would otherwise be unable to attend an in-person intervention [17]. Encouragingly, these advantages were also emphasized by caregivers in this study. Although most caregivers felt that the text messages were acceptable and beneficial, one of the top disadvantages mentioned in the focus group was that some caregivers might not have text messaging–capable devices. This perception is inconsistent with a recent report from 2018 that found 91% of rural residents had access to mobile phones [16]. This discrepancy may be because of the fact that mobile phone ownership has risen dramatically in the past few years [16], and the caregivers in this study may not fully understand the rates of cell phone ownership in their communities. Other disadvantages mentioned included poor coverage in very rural areas and the use of temporary phones. Despite these disadvantages, caregivers felt text messages could be effective and beneficial in their respective communities. Collectively, the text message advantages accentuated in the focus groups outweighed the disadvantages. Given the potential continued increase in the use of text messaging technology in rural populations and the benefits it provides to overcoming barriers to accessing evidence-based programs, this is an optimal time to develop and test text message–based interventions.

### Language Preferences

As technology for delivering health behavior interventions advances, theoretical approaches must continue to be utilized. Few studies have documented the development and testing process

for text messages using theory-based approaches [17], particularly those that focus on the features of language. Linguistic theory postulates that word choice and underlying tones can help the target audience identify with the messages and, in conjunction with behavior change theories, can produce desired health outcomes [25]. Furthermore, theorists have stated that considering cultural perspectives of the targeted population when developing health education messages could lead to not only an appreciation of the messages but ultimately effectiveness and adoption of the desired intervention [35].

Results from the focus groups revealed several important language considerations for the targeted rural caregiver population and are also supported by a study by Denham et al around health messaging for Appalachian residents [36]. Overall catchy type tones were disliked because of the use of slang and trendy words that some caregivers found unappealing, yet the memorable aspect of these messages was liked. Authoritative tones were preferred, as long as the messages were providing useful strategies and stayed away from absolute words (ie, always, never, and only). Empathetic tones were also liked, as long as the messages were not making assumptions about the caregivers’ SSB intake behaviors or using condescending tones. Finally, although no audience preferences emerged from the card-sorting activity, the benefit of a family-based approach was a prominent theme that emerged from focus group discussions and post interviews. Importantly, Denham et al found similar results when conducting focus groups around health messaging to decrease underage drinking and tobacco use, though not exclusive to text messages. Their study suggests that messages should be based on fact, have a polite tone, and present information in a nonjudgmental way; findings that align with the results presented here. Furthermore, this same study found the importance of family-based approach, particularly among women who felt they were the gatekeepers to their family’s health [36]. Together these results suggest that preferences for message language and framing is consistent between delivery modalities and health behaviors and provide a strong foundation of evidence for future health messaging development in rural and Appalachian areas.

### Text Message Use and Preliminary Effectiveness

During the text message pilot trial, caregivers interacted with the messages by responding to assessments, making mental notes, and sharing messages with family and friends. The high rate with which caregivers utilized the text messages in this study indicates that this may be an effective modality for caregivers to receive SSB intake–related behavior change strategies. This finding is supported by several studies that have found greater adherence to self-monitoring practices and higher intervention completion rates through text message use [19,37]. Not only does text messaging have the potential to increase self-monitoring adherence, results in this study suggest that text messages delivered to caregivers may also be an effective method to improve caregiver SSB intake–related behaviors and reduce SSB intake of both caregivers and adolescents. Studies have found that parenting practices, home environment, and parental role modeling have significant influence over adolescent SSB intake [31,38], but few studies have studied this using text messages. A study by Grutzmacher et al found that delivering a nutrition and physical activity intervention through text messages to low-income parents of school-aged children significantly improved home environment, parent behaviors and intake, and child intake around fruit and vegetable consumption [39]. Findings from Grutamzcher et al reinforce this study’s preliminary results and emphasize the potential for text messages delivered to caregivers to change caregiver and child health behaviors.

### Limitations

The results of this study should be interpreted in the context of several limitations. The recruitment methods may have resulted in a more motivated and informed sample of caregivers. Caregivers who are less motivated to change their SSB intake may have had different reactions to the language and content of the messages. However, during the focus group elicitation process, caregivers were asked to think about themselves and their whole community. Also, this study was over representative of females, high income, high education, and lower SSB intake. SSB intake in this study sample was lower than what has been found in previous research with representative samples [40]. These factors may limit the generalizability of the results.

In addition, there are several limitations in interpreting behavior change results. First, although validated measures were used, self-reported behaviors by the caregiver and may have introduced bias or human error. Second, it is important to distinguish that the tested text message strategy was not a stand-alone intervention. The pilot trial was preceded by a focus group, so caregivers naturally received some education and face-to-face discussion around SSBs before receiving messages. This makes it difficult to tease out the isolated effects of the pilot trial. Finally, this pilot study did not compare behavior change to a control group and the pre-post changes should be interpreted somewhat cautiously because of the small sample size. Despite these limitations, the effect sizes provide general estimates to inform fully powered future studies and a framework for future study with text message–based health interventions in rural areas.

### Conclusions and Future Directions

This study aimed to develop and test relevant text messages to reduce the high consumption of SSBs in rural areas, which may be contributing to the widespread health disparities. Spending time in the formative phases of text message development helped understand the unique perspectives and language preferences of the target population. This study also found that delivering an intervention through text messages had the potential to reduce SSB intake in rural caregivers and adolescents, where SSB intake is a prevalent problem [9,40]. This is promising because text message has many benefits, such as being low-cost, easily accessible, and asynchronous, which may help overcome some of the current barriers to programming faced by rural populations.

This text message–based intervention to reduce SSB consumption in caregiver and adolescents shows promise, yet there are important considerations for future study. Fully powered studies are needed to determine engagement, adherence, and effects of a text message intervention targeting SSBs. Future text message studies in rural areas should aim to incorporate the ability to choose the frequency and timing of the delivery of messages to fit the needs of various caregivers’ work schedules and general preferences for increased adherence and response rates. In addition, future studies should explore incorporating tailored feedback, as studies have shown that they improve behavioral outcomes [20,41]. Finally, studies should be conducted to understand how text messages can be used or incorporated into interventions that target multiple levels of influence on caregiver and adolescent SSB intake behaviors [42].

## Appendix

Multimedia Appendix 1 A short video demonstrating how caregivers received and interacted with the text message intervention.

Multimedia Appendix 2 Results from semistructured focus group discussion with representative quotes around using text messages for sugar-sweetened beverage behavior change.

## Abbreviations

BEVQ-15: 15-item beverage intake questionnaire
NIH: National Institutes of Health
SSB: sugar-sweetened beverage
