# Supplementary material for: Development and Pilot Testing of Text Messages to Help Reduce Sugar-Sweetened Beverage Intake Among Rural Caregivers and Adolescents: Mixed Methods Study
Source: JMIR Mhealth Uhealth. 2019 Jul 30;7(7):e14785. doi: 10.2196/14785 (PMC6691674; doi:10.2196/14785)
Supplement: Multimedia Appendix 2 [file mhealth_v7i7e14785_app2.pdf]

Multimedia appendix 2. Results from semistructured focus group discussion with representative quotes around using text messages for sugar-sweetened beverage behavior change**.**

Category and codes		Definitions	Representative quotes
**Advantages of using text messages for SSB[a] intake behavior change**			
	Convenient/good timing	Participants feel that text messaging is a convenient method of sending information because of timing.	"I think one advantage is that they can look at it on their own time. If it comes through at a bad time, then I can look at it later and think about it when it is convenient."
	More accessible	Participants stated that they had their phone on them all the time or it was more accessible than other means of communication.	"You'll get right to them...I mean they've got their phone on them."; "I'm thinking of it as opposed to various in comparison to like a flier that would be in a school notebook. It may not get lost because then you would see it more regularly I guess."
	Easy to read, reread, and understand	Participants felt that messages were a simple, easy method to send information because they were short and easy to understand. They can also be saved and reread.	"Parents don't wanna take a lot of time. They don't wanna take you know, we even have parents who can't read very well. I mean, you know so... that would be simple for them instead of, you know, [a handout] or a big long email."; "If you send me a text, most likely I'll have a phone and most likely I can reread that sucker if I need to reread it. For me that's the reason I like text better"
	Most plans support text messages	Participants stated that most cell phone plans included a text messaging component, an unlimited text messaging component, or only a few percentages of people would not have text messaging plans.	"Nobody complains when I send a text, so..."; "I think the texts are pretty much unlimited...It's probably data that would be the issue."
	Quick response	Participants stated that they would respond much more quickly to a text message than to other forms of communication.	"I know you'll get me faster if you send me a text than you will an email."
	More reach	Participants felt that more people would be reached through sending text messages compared with other forms of communication.	"You'd be including more people. Most people have a cell phone now. I'm one of the few who still has a landline in their house."

**Disadvantages of using text messages for SSB intake behavior change**			
	No text message–capable devices	Participants felt that people in the region might not have text message capable–devices.	"Well I guess there could be some that wouldn't have the technology, they wouldn't have a cell phone. Depending on where you are."
	Poor coverage/service areas	Participants reported that some areas in the region would have poor reception, coverage, or service.	"There's still places that don't [have service]. I know a friend of mine that has to wait till she comes up the road."; "I lived up in [redacted]. You'd have to sit out on the porch with your arm like that. When they did have service out there you wasn't getting none."
	Temporary phones/numbers	Participants stated that people in this region might have temporary phones and phone numbers that changed often.	"We know that from trying to get ahold of parents. They may have one phone and the next month that number doesn't work anymore. It's very difficult."
	Can be repetitive/people can easily ignore	Participants felt that receiving text messages about the same behavior every week might be repetitive and it could be easy for people to ignore.	"...there's probably a too often you know that people will tune it out, but I don't know what that cutoff is."
	Plans have limited text messages	Participants stated that cell phones might not have an unlimited text messaging component or might get charged for receiving text messages.	"And I wonder, does everybody automatically have unlimited texting? I don't know if that's something."
**Liked language and features of text messages**			
	Memorable phrases	Participants liked messages that contained phrases they themselves and their children could remember and repeat.	"It rhymed and it would be easy to remember if you were going over it with your family. That was my theory on that."; "It's like a little rhyme...I think it's something that the kids would repeat, too."
	Family approach	Participants liked messages and phrases that targeted a family approach for drinking less SSBs.	"And I think that as a mom, we think more of the family as opposed to one child. Does that make sense? Because since you're targeting middle school, you may have younger kids and you may have older kids, whereas if you're targeting a family you can change everybody's, and then you're helping all age groups."
	Provides useful information	Participants liked messages that provided useful advice, information, and solutions to drinking less SSB.	"Yeah. It gives you clear cut instructions....Some things you think it doesn't have sugar, but it teaches you'll

			always look for the label and if it says zero then it's [ok]."
	Telling you what to do with strategies	Participants liked messages that told them what to do as long as they were telling them what strategies to try.	"I think I would like to see more tips for how, how to reduce. Like give me some ideas. I might not know where to start."
	Sparks discussion with family	Participant liked messages that had a call to action that included changing behaviors of or starting discussion with family members.	"...for younger kids it's a way for you to know what they're doing in school, and maybe a game to play with them to just reinforce those concepts."; "And maybe getting them involved in the process of it. Informing the child or informing your family this is what you need to be looking for in order to make a healthy choice."
	Encouraging	Participants liked encouraging messages.	"I guess I liked the fact that you're saying yeah, we get that it's hard to, but here's an alternative. You're not just saying 'stop' or 'don't do it', you're saying 'we understand it's hard, but try this'."; "'Keep reducing' kind of sounds like 'hey, you're doing it! You're doing it! Just keep going'."
	Honest/truthfulness	Participants liked messages that were honest, truthful, to the point, and without any *fluff*.	"The truth about [companies] using the pics and slogans to make us buy their products. I think that is the truth."
**Disliked language and features of text messages**			
	Telling you what to do	Participants disliked informational messages that told them what to do without giving useful strategies to help change that behavior.	"...if you tell somebody to stop doing something, that's what they're gonna do. They're just gonna... [do] the opposite."; "..they'll just skip it. You know if you're telling me what to do, I'm not going to even read the rest of that, so."
	Judging	Participants disliked messages that were judging them as a parent or insinuating poor parenting.	"I looked more at 'don't slack, [cut back!]'. Well just because I give my kids PowerAde, doesn't mean I'm a slacking parent. Slacker to me means negative. And we are working as hard as we can to be the best parent we can be. And you're saying if I give my kid a sugary drink every now and then, I'm slacking."
	Use of symbols	Participants disliked messages that used symbols, such as greater than sign or an exclamation point, because they felt people	"I think the greater sign, some may not understand at all."

		might not understand their meaning or purpose.	
	Condescending/demeaning tones	Participants disliked messages that had condescending and demeaning tones.	"I think tricky has been a word that has been overused a lot. It's condescending that's questioning intelligence. Questioning like you're just zombies zoned into TV and not fully putting any thought into anything."
	Assumptions/ making excuses	Participants disliked messages that made assumptions about their SSB intake habits or made excuses for their SSB intake habits.	"I'm not a victim of excuses. You just do it or you don't. It's not that it's hard. You just do it."; "The thing I didn't like about it was, like 'we know it's hard to cut back'. Like that sounds a little bit presumptuous. Why is it hard to cut back?"
	Use of absolute words	Participants disliked words, such as always, never, or only that represented an absolute or unbounded period of time.	"Mine was the 'the labels always tell the truth'. That was something if I read that I would be skeptical automatically when I saw that 'always tells the truth'. Because I guess that one of the words that you always look for. When you see always or never you never choose those."
	Blame displacement	Participants disliked messages that placed blame on companies or other entities for their adolescent's SSB intake behaviors.	"That's why they are in business. I don't see that as a problem. I just don't like the idea of like she mentioned blaming McDonalds...it's their fault that I eat too many french fries. No, it's my fault that I eat too many... I don't like blaming people for what I do."
	Use of slang or trendy words	Participants disliked the use of slang or trendy words, such as *fam* or *nah*.	"I don't like the slang. Just because we're Appalachian doesn't mean we're all hicks. I took that as a negative."
	Use of governmental or official words	Participants disliked use of governmental or official words, such as *research*.	"Where it says research, for someone who values research, or thinks about research frequently, that may make a difference, thinking about wow okay, that makes sense, or I didn't realize that, whereas someone with a low, lower health literacy, it might be like 'I don't care'... I think it would turn them off. "
**Personalization of text messages**			
	Grabs attention (positive)	Participants thought personalizing messages with adolescent/parent names grabbed attention.	"...everybody loves to hear their name. If the parent's first name is on there, might draw them in more."
	Unnecessary (negative)	Participants felt that personalizing with names	"I don't have to have a name. I just want you to tell me what you want to tell me and

		was unnecessary and/or would not make a difference.	move it on. And then I'll read your message. If it's globbity glook I'm not going to read it probably. That's just the truth."
**Completing assessments via text messages**			
	Helps with reaching goals (positive)	Participants felt that assessments would be beneficial in helping them keep track of and reach their goals.	"I mean, if you text me and I say what's at my house at the time, the likelihood is I'm gonna look around and see what's in the house and if I have a goal then I'm gonna work on decreasing it."
	Poor response rates/unreliable responses (negative)	Participants felt that assessments might have poor response rates or that people might not be truthful in their reporting.	"...there's also some of these people may get into this initially and then they may just tell you what you wanna hear just to get you...get an answer to you."
	Increase accountability (positive)	Participants felt that assessments would be beneficial in increasing accountability for their goals.	"[It's] like having an accountability group or somebody that's checking up on you."
	May be too lengthy/overwhelming (negative)	Participants felt that assessments might be too lengthy, and therefore, disliked by caregivers.	"Well don't do it a lot or it will get on my nerves, but if you ask me one question I will probably answer you."
**Preferred timing for text messages**			
	While caregiver is with adolescent/after school	Participants felt receiving messages while with adolescent would be most beneficial.	"I like to send em right after school cause their kids are on the way home and... they're gonna say 'they've got this to do, this to do, all games'."
	Start of the month/week/day	Participants felt receiving messages at the start of the month or the week would be beneficial because that was when people might plan and set goals.	"Yeah I don't have any worries yet in the morning. And if by 3 or 4 o'clock I've had a couple stresses probably that are taking up my mind, then a Coke is going to be fine, but in the morning, I might set a goal for water."
**Preferred frequency of text messages**			
	1-2 times/week	Participants preferred receiving messages 1-2 time per week.	"1 to 2 times a week...If you do more than that they aren't going to respond."

[a]SSB: sugar-sweetened beverage.
